# Inter-annual variations of vegetation dynamics to climate change in Ordos, Inner Mongolia, China

**DOI:** 10.1371/journal.pone.0264263

**Published:** 2022-11-04

**Authors:** Xiru Jia, Guangyong You, Shawn McKenzie, Changxin Zou, Jixi Gao, Anlan Wang

**Affiliations:** 1 School of Environment & Natural Resources, Renmin University of China, Beijing, 100872, China; 2 Nanjing Institute of Environmental Sciences, Ministry of Ecology and Environment, Nanjing, Jiangsu, 210042, China; 3 School of Earth, Environment, and Society and McMaster Centre for Climate Change, McMaster University, Hamilton, Ontario, Canada; 4 Ministry of Ecology and Environment Center for Satellite Application on Ecology and Environment, Beijing, 100094, China; 5 School of Remote Sensing and Geomatics Engineering, Nanjing University of Information Science and Technology, Nanjing, Jiangsu, 210044, China; Chinese Academy of Forestry, CHINA

## Abstract

To reveal the characteristics of climate change and the controlling factors for vegetation dynamics in the Ordos, Inner Mongolia, China, 34 years (1982–2015) of regional climate variables and vegetation dynamics were investigated. The results show that: Annual mean air temperature (TMP) significantly increased with a linear slope of 0.473°C/10yr. Annual precipitation (PRE) had a non-significant positive trend nearly 5 times lower than the trend of potential evapotranspiration (PET). The average Normalized Difference Vegetation Index (NDVI) computed for the region was found to show a significant positive trend (6.131×10^−4^/yr). However, all climate variables displayed non-significant correlations with NDVI at annual scale. The reduction of desert and the increase of grassland over the past decades were accountable for the increased NDVI. Principal components analysis revealed that the regional climate change can be characterized as changes in temperature, humidity and the availability of radiant energy. Based on principal components regression coefficients, NDVI was mostly sensitive to humidity component, followed by growing season warmth (WMI). Spatially, 93.1% of the pixels displayed positive trend and 61.8% of the pixels displayed significant change over the past decades. Both principal regression analysis and partial correlation analysis revealed that NDVI in eastern part of Ordos was sensitive to TMP, whereas, NDVI in southern and western areas of Ordos displayed the high sensitivity to combined effects of PRE and cloud coverage (CLD). Partial correlation analyses also revealed that TMX was a surrogate for aridity, TMN was a representative of humidity, and temperature variations below the threshold of 5°C (CDI) were less important than WMI. We conclude that regional climate change can be characterized by warming and increased aridity. The significant positive trend of regional NDVI and the non-significant correlations between NDVI and climate variables at annual scale suggests the hidden role of the human activities.

## 1.Introduction

Vegetation is a critical component of the terrestrial biosphere and its growth activity plays an important role in the global carbon and hydrological cycles [1]. Studies have shown that there are clear relationships between climate data and Global Inventory Modeling and Mapping Studies (GIMMS) satellite-derived normalized difference vegetation index (NDVI) vegetation dynamics [2]. Additionally, feedbacks have been revealed between vegetation activity and the carbon and hydrological cycles [3–7].

Most of these studies concentrated exclusively on empirical data such as observed temperature and precipitation. Generally, these studies do not consider derived climate variables such as evapotranspiration [8], cloud cover [9], and warmth/coldness indices [10]. In addition, the effects of asymmetrical climate change, which is characterized as a higher increase of minimum temperatures and a smaller increase in maximum temperatures, on vegetation growth have not been sufficiently investigated [11,12].

Traditionally, studies that have identified significant climate-vegetation relationship with linear correlation analytical approach [13]. Limited consideration has been given to the co-linearity among climate variables, which may results in pseudo driving factors for vegetation dynamics [14,15].

Moreover, it is challenging to differentiate between the climate and human causations on vegetation dynamics. Urbanization, cropland expansion, demographic changes, and livestock grazing activity have been found to impact climate-vegetation dynamics, especially within the forest-steppe-desert transition zone where different trends and driving factors coexist [6,16,17]. Traditional linear correlation approaches provide an incomplete representation of variable-vegetation analysis, which may lead to biased conclusions on the scope of climate impacts on vegetation and thereby influence regional climate-vegetation modelling scenarios. For example, the expansion of cropland in north-eastern Inner Mongolia misled our understanding about the climate-vegetation relationship [10]. A climate-vegetation study in southern Africa underestimated the severity of land degradation using the linear regression method [18].

The Ordos Plateau of Inner Mongolia, China, is a typical forest-steppe-desert transition zone. The ecosystem diversity and vegetation dynamics in this region are highly sensitive to both climate change and anthropogenic influences [19]. These climate-vegetation relationships are complicated with asymmetric climate change and human activities that take place over the past three decades. These relationships are further complicated by numerous national and local ecological restoration projects that had been implemented by the government since the year 2000 [20,21]. Therefore, investigations on the climatic variations and human activities on regional vegetation changes are critical to characterize the vegetation growth responses to climate change. As underlying causes are identified in a robust manner, further progress can be made on the restoration and reconstruction policies of the Ordos Plateau ecosystems, which could be expanded into the vicinity regions with similar climate settings.

In this study, climate datasets were compared to vegetation dynamics in the Ordos Plateau over a 34-year period (1982–2015) to evaluate the regional climate-vegetation relationship. This study aims to i) characterize the temporal patterns during the regional climate change, ii) evaluate spatial patterns of vegetation-climate relationships, and iii) identify the controlling factors that drive vegetation dynamics in the Ordos ecosystems.

## Materials and methods

### Study site

The Ordos region is located in the southern part of the Inner Mongolia Autonomous Region (37°41′-40°51′N; 106°42′-111°31′E) and occupies approximately 87,400 km^2^ [22]. The area is bounded on the north by the Hobq Desert and the south by the Mu Us Sandy Land [22,23]. The major ecosystems are grassland, sandy land, cropland, meadows and forested land [22,24].

The study site experiences a temperate continental climate with high evaporative demand during the growing season, which typically lasts from May to October. The mean annual temperature is 5.3–8.7°C with a high of 23.5°C during the hottest month (July) and a low of -11.5°C during the coldest month (January). The total annual precipitation is 150–450 mm with more than 70% of the total annual precipitation occurring from July to September. Generally, air temperature declines with elevation, whereas, precipitation decreases from east to west across the region [13]. Geologically, the region is sub-divided into the northern Yellow River alluvial plain, the eastern hilly region, the western arid plateau and the southern sandy plain; these geological features influence the vegetation types and the strength of aridity.

### Data collection

To capture the temporal dynamics of vegetation change in response to climate change, 34 years (1982–2015) of Normalized Difference Vegetation Index (NDVI) of Ordos were obtained from the Global Inventory Monitoring and Modeling System (GIMMS) project (https://ecocast.arc.nasa.gov/data/pub/gimms/, accessed at 10/Oct/2018). To account for the issues of orbital drift, sensor degradation and radiation effect of volcanic eruption, GIMMS NDVI3g data have been normalized to produce a non-stationary 1981–2012 AVHRR NDVI3g time series [25]. The spatial resolution of the data was at 1/12° and pixels with low quality data (flag value = 4–7) were removed from the analysis [26].

To reveal the characteristics of regional climate change, climate datasets (Table 1) were obtained from the Climatic Research Unit (CRU: University of East Anglia Climatic Research Unit, UK) data archives [27]. These CRU datasets contain homogenized monthly time series of precipitation, daily maximum and minimum temperatures, cloud cover, and other variables, all of which were gridded to 0.5x0.5 degree resolution. To match the spatial-temporal resolutions of GIMMS ndvi3g and facilitate the calculation at pixel scale, the climate datasets (0.5°×0.5° resolution) were disaggregated into 1/12° by bilinear interpolation of the neighboring pixels.

**Table 1:** Abbreviations and data sources for the climate variables and NDVI.

Abbreviation	Variables	Unit	Source/Method
TMP	Daily mean temperature	°C	CRU TS v4.01
TMN	Daily minimum temperature	°C	CRU TS v4.01
TMX	Daily maximum temperature	°C	CRU TS v4.01
WMI	Warmth index	°C·month	(Kira, T., 1945)
CDI	Coldness index	°C·month	(Kira, T., 1945)
PRE	Precipitation	mm	CRU TS v4.01
PET	Potential evapotranspiration	mm	CRU TS v3.23
VAP	Vapour pressure	hPa	CRU TS v4.01
CLD	Cloud coverage	%	CRU TS v4.01
NDVI	Normalized Difference Vegetation Index	-	GIMMS ndvi3g

As warmth index (WMI) and coldness index (CDI) were effective indicotors for vegetation growth and distribution limits [10,15,28], we followed You et al. [10] to established annual anomalies of WMI and CDI at each pixel by counting the annual sum of positive and negative differences between monthly means and 5°C [29].

To obtain further information on vegetation trends, MODIS land cover product (MCD12Q1) for 2001 and 2015 were collected from https://e4ftl01.cr.usgs.gov/MOTA/MCD12Q1.006/. The definition of land use types followed the International Geosphere-Biosphere Program (IGBP) land cover classification system, and the accuracy of the land cover dataset has been validated by field observations [30].

### Analytical methods

To reveal the long-term trends of climate variables and NDVI, both non-parametric trend tests and linear trend tests were conducted [31,32]. To reduce the influence of autocorrelation on trend detection, the datasets were pre-whitened (MK-TFPW) prior to applying the Mann-Kendall trend test [33]. To reduce the influence of abnormal anomalies and outliers on the results of the trend analyses, the non-parametric Sen [34] slope was also obtained by calculating the median slope between all pairwise combinations of points in time series of climate variables and NDVI. To reveal any significant change points within time series of climate variables and NDVI, the Mann–Whitney–Pettit test was conducted [35]. Detailed time series analyses and correlation analyses were presented by You et al. [10].

To remove the impact of co-linearity between the climate variables, principal components regression (PCR) was used to identify the relative importance of each variable driving interannual variations of NDVI [9,10,15]. In each pixel, we first extract 3 principal components from the climate dataset, representing temperature, humidity and global radiation components. Then, we established multi-linear regression model by using standardized NDVI as dependent and using the loading scores of the 3 components as independents. Then, we multiplied the loadings of each variable by the aforementioned multi-linear regression model coefficients and summed these scores. This enabled us to estimate the relative importance of each variable in driving the interannual variations of NDVI. For the annual means of regional climate variables and NDVI values, we extract 6 principal components representing more than 99% of variance of the climate dataset.

The partial correlation was conducted to reveal the correlation between X and Y on condition of the effect due to the third variable Z was eliminated (Eq 1). Suppose we are given three variables X_1_, X_2_ and X_3_. Let r_12_ denote the correlation coefficient between the variables X_1_ and X_2_. Then the partial correlation coefficient between X_1_ and X_2_ by controlling for the effect of X_3_ is denoted by the symbol r_12,3_. It is given by the following formula:

$$r_{12,3}=\frac{r_{12}-r_{13}r_{23}}{\sqrt{\left(1-r_{13}^{2})(1-r_{23}^{2}\right)}}$$ (1)

## Results

### Trend analyses

**Fig 1:**
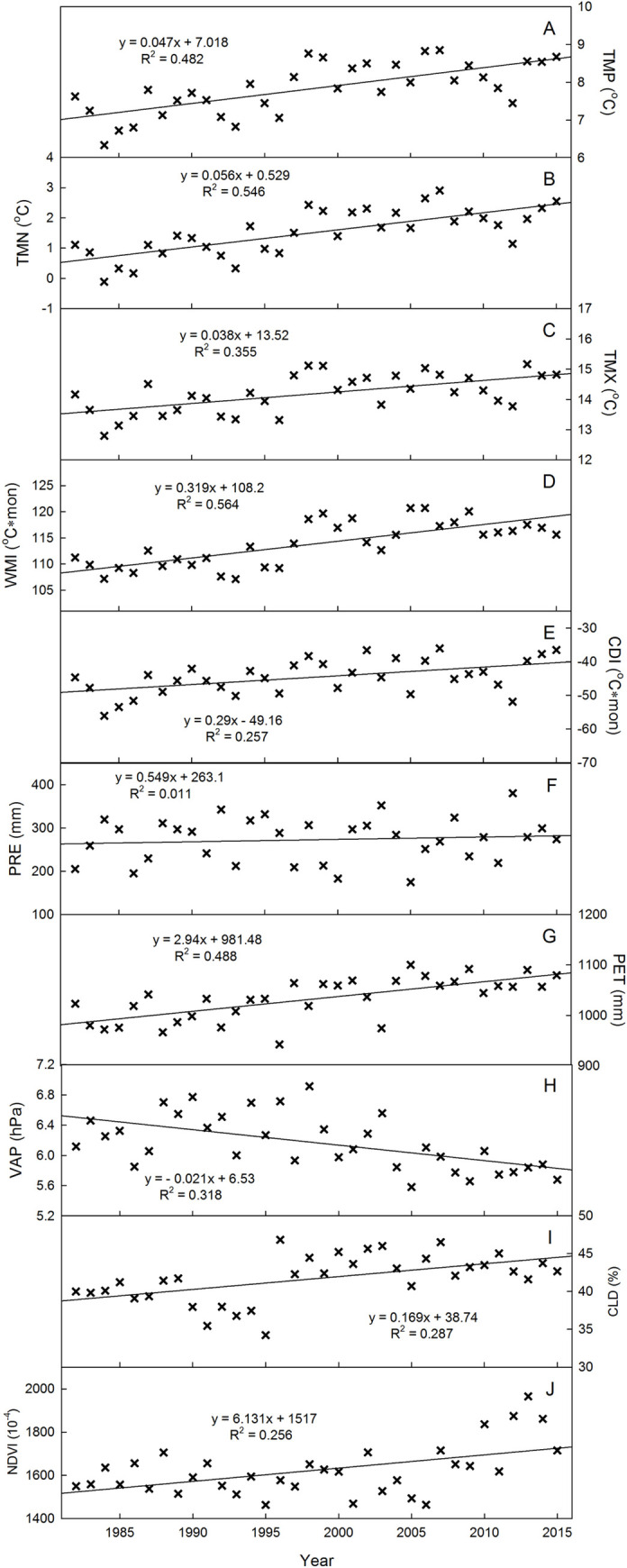
The annual anomalies and linear slopes of A) mean annual temperature (TMP), B) mean annual daily minimum temperature (TMN), C) mean annual daily maximum temperature (TMX), D) warmth index (WMI), E) coldness index (CDI), F) total annual precipitation (PRE), G) total annual potential evapotranspiration (PET), H) mean annual vapor pressure (VAP), I) mean annual cloud coverage (CLD), J) mean annual Normalized Difference Vegetation Index (NDVI).

Regional climate change and vegetation dynamics for the Ordos region during the 1982–2015 study interval are displayed in Table 2 and Fig 1. Significant change points were detected during the year 1996–1997 in all of the temperature-related and cloud cover variables (TMP, TMX, TMN, WMI, CDI and CLD). Temperature-related variables displayed an increasing inter-annual trend prior to the 1996 change point (TMP, TMX, WMI and CDI). These variables then displayed a negative trend after 1996. At decadal scales, TMP showed an increasing slope (0.473⁰C/10yr) (Fig 1A), but the increase was not significant. Prior to the change point, TMN significantly increased (0.567⁰C/10yr; p<0.01) (Fig 1B), while TMX increased but was nonsignificant (0.473⁰C/10yr) (Fig 1C). These two trend trajectories suggest that asymmetric warming had occurred and that minimum temperatures were rising faster than maximum temperatures.

**Table 2:** Time series analyses of climatic variables and NDVI over the past three decades (1982–2015).

Climate Variables	MK-tau	Pwmk tau	Sen slope (10 yr^-1^)	Linear slope (10 yr^-1^)	Change point year	Linear slope (1982-point, 10yr^-1^)	Linear slope (point-2015, 10yr^-1^)
TMP (°C)	0.494**	0.261*	0.472	0.473	1996**	0.239	-0.036
TMN (°C)	0.522**	0.258*	0.582	0.567**	1996**	0.36	0.071
TMX (°C)	0.390**	0.250*	0.384	0.380**	1996**	0.12	-0.142
WMI (°C·month)	0.490**	0.170	3.033	3.196**	1997**	0.938	-0.637
CDI (°C·month)	0.412**	0.295*	2.894	2.931**	1996**	4.464*	-0.31
PRE (mm)	0.030	-0.030	2.286	5.495	1987	-27.02	-2.092
PET (mm)	0.494**	0.345**	28.966	29.397**	1996	2.205	15.95
VAP (hPa)	-0.398**	-0.314*	-0.2	-0.205**	2003**	0.033	-0.058
CLD (%)	0.348**	0.216	0.145	1.697**	1995**	-3.870**	-0.903
NDVI (×10^−4^)	0.269*	0.152	45.51	61.31**	2006**	-21.01	323.1*

*. Correlation is significant at the 0.05 level (2-tailed).

**. Correlation is significant at the 0.01 level (2-tailed).

PRE displayed a nonsignificant positive trend (5.5 mm/10yr; nonsig.) (Fig 1F). Conversely, PET displayed a significant positive trend (29.4 mm/10yr; p<0.01) (Fig 1G), which suggests increased aridity took place in the region. As a consequence, VAP trends significantly decreased (-0.205 hPa/10yr; p<0.01) with a significant change point in the year 2003 (Fig 1H). The regional NDVI displayed a significant positive trend (6.131×10^−3^/10yr; p<0.05) with a significant change point in the year 2006 (Fig 1J). Prior to the change point, NDVI declined from 1982–2006. After 2006, NDVI increased significantly.

### Principal component analysis

For the inter-annual means of climate variables, principal component analysis shows the 1st principal component of the 9 climate variables was highly correlated with TMP, TMN, TMX, WMI, CDI and PET; all of which represent the temperature component (Table 3). The 2nd principal component was highly correlated with PRE and VAP, which together represent the humidity component. The 3rd principal component was highly correlated with CLD, which representing the solar radiation component. Therefore, regional climate change can be summarized as the changes in temperature, humidity and the availability of radiant energy.

**Table 3:** Correlation coefficients between climate variables and the principal components. PRC is principal regression coefficients of NDVI against the climate variables. NDVI columns are the Pearson and Spearman correlation coefficients between NDVI and climate variables.

Climate variables	Principal Components							PRC	NDVI	
	PC1	PC2	PC3	PC4	PC5	PC6	PC7		Pearson	Spearman
TMP	0.962	0.199	-0.107	0.092	-0.069	-0.093	0.038	-0.053	0.242	0.258
TMN	0.933	0.303	-0.014	0.030	-0.034	-0.045	0.183	0.022	0.234	0.263
TMX	0.934	0.062	-0.210	0.162	-0.107	-0.146	-0.140	-0.142	0.237	0.226
WMI	0.919	-0.029	0.167	-0.054	-0.267	0.222	-0.022	0.194	0.230	0.187
CDI	0.781	0.243	-0.314	-0.007	0.466	0.119	-0.031	0.100	0.189	0.177
PET	0.860	-0.429	-0.118	-0.202	-0.111	0.026	-0.026	0.114	0.239	0.127
VAP	-0.489	0.705	-0.196	0.433	-0.171	0.087	-0.021	-0.197	-0.245	-0.223
PRE	-0.217	0.754	-0.081	-0.607	-0.088	-0.027	-0.034	0.326	0.222	0.137
CLD	0.515	0.353	0.761	0.082	0.140	-0.030	-0.051	0.094	0.244	0.248
Cumulative variance	0.601	0.776	0.867	0.938	0.980	0.992	0.998	-	-	-

Correlation coefficient ≥ 0.349 means 0.05 level (2-tailed) significance.

Correlation coefficient ≥ 0.448 means 0.01 level (2-tailed) significance.

For the inter-annual means of NDVI, the correlation analyses showed that all climate variables displayed non-significant correlations with NDVI (Table 3). This lack of correlation suggests regional vegetation dynamics were not controlled by individual climate variables exclusively. The principal regression coefficients (PRC) show that NDVI was mostly sensitive to humidity component, followed by WMI. This sensitivity indicates humidity and growing season warmth were important control factors on the inter-annual variation of NDVI.

### Spatial distribution of NDVI trend

**Fig 2:**
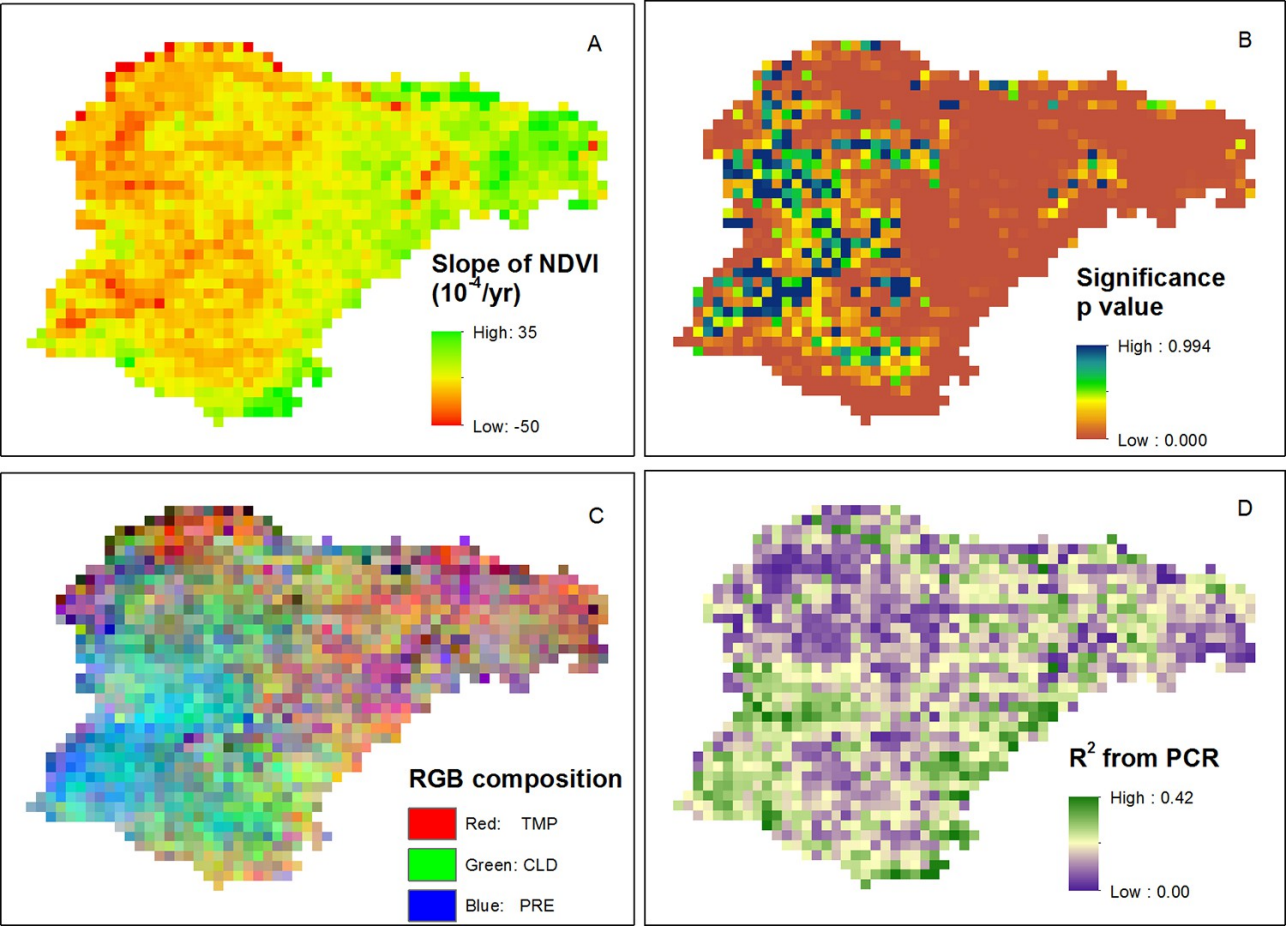
Pixilation trends in the Ordos study area: A) Spatial distribution of NDVI slope, B) significance of NDVI trend, C) color composite of vegetation sensitivity to TMP (red), PRE (blue) and CLD (green), and D) R^2^ values of the principal regression analysis.

The majority of the pixels (93.1%) displayed a positive trend in NDVI during 1982–2015 (Fig 2A). Additionally, most of the pixels (61.8%) displayed a significant change in the trend of NDVI over the past decades (Fig 2B). Principal regression analyses revealed that the change in vegetation in the eastern part of Ordos was sensitive to TMP. In the southern and western areas of Ordos, vegetation change was sensitive to the combined effects of PRE and CLD (Fig 2C). Overall, the R^2^ from principal regression analysis, residuals of climate-vegetation relationship, was low, suggesting the weak link between climate and vegetation and the hidden role of anthropogenic influences (Fig 2D).

### Land use and land cover changes

The land use and land cover transition matrix from 2001 to 2015 displayed that the groundcover area of grasslands, shrubland, urban lands and croplands had increased from 2001 to 2015 (Table 4). Remarkable changes are the the increase of grassland and the decrease of desert land. Additionally, this analysis showed that 21.1% of the total desert area in 2001 was replaced by grassland in 2015.

**Table 4:** The Land use (MODIS MCD12Q1) transition matrix of Ordos from 2001 to 2015 (km^2^).

2015 2001	Desert	Cropland	Grassland	Shrubland	Urban	Wetland	Unknown	SUM (2001)
Desert	10047	2	2691	3	1	0	0	12745
Cropland	7	584	174	0	0	0	0	765
Grassland	194	239	73315	33	8	2	0	73791
Shrubland	0	0	3	0	0	0	0	3
Urban	0	0	0	0	86	0	0	86
Wetland	0	0	1	0	0	0	0	1
Unknown	2	0	1	0	0	0	8	10
SUM (2015)	10250	825	76184	36	95	2	8	87400

### Partial correlation analysis

**Fig 3:**
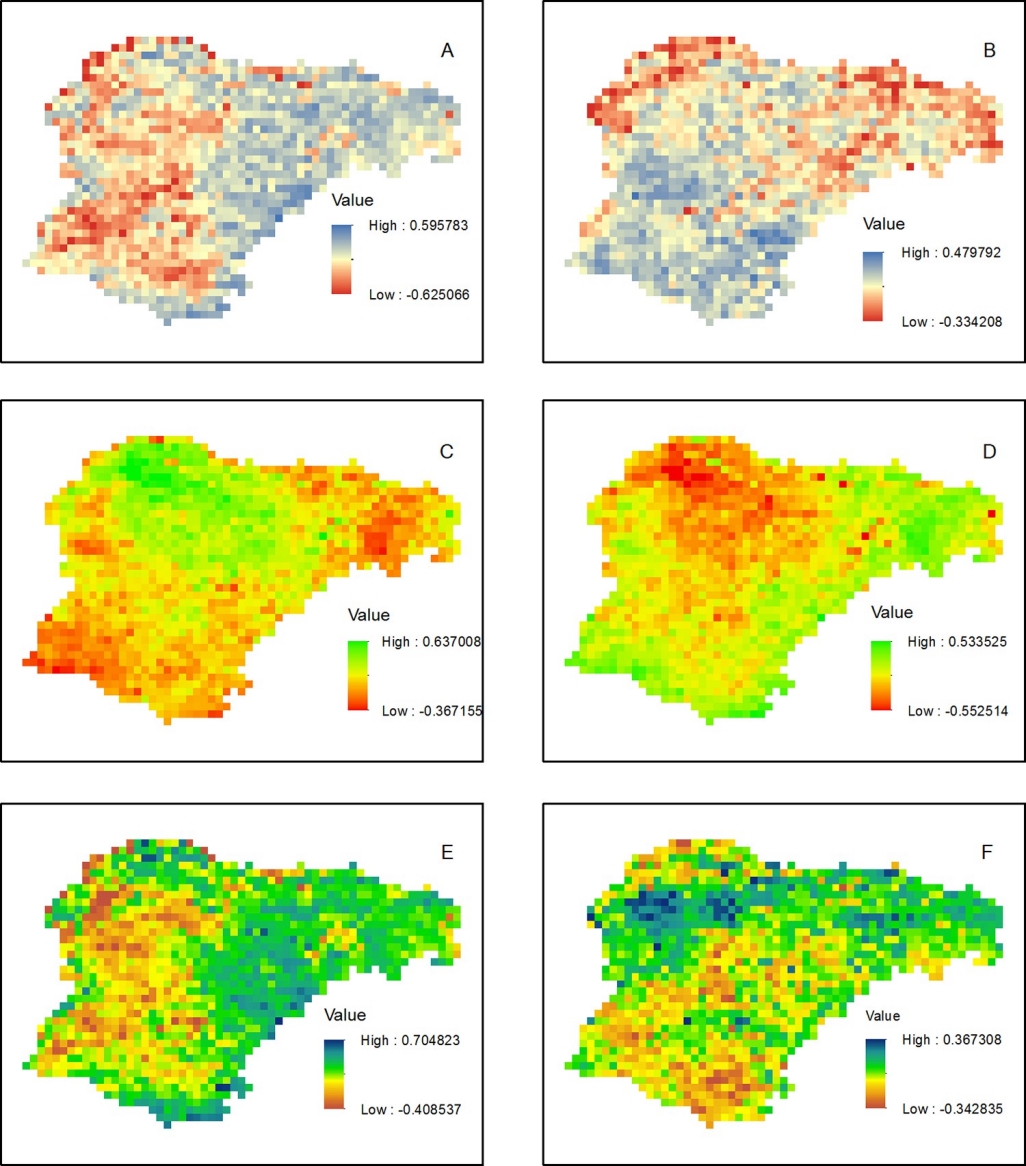
Spatial distribution of partial correction coefficients between NDVI and TMP (A, controlled PRE), NDVI and PRE (B, controlled TMP), NDVI and TMX (C, controlled TMN), NDVI and TMN (D, controlled TMX), NDVI and WMI (E, controlled CDI), NDVI and CDI (F, controlled WMI). The significance at 0.05 probability level is ±0.349.

Partial correlation analyses were conducted to reveal the comparative roles of individual climate variables (TMP vs. PRE, TMN vs. TMX, WMI vs. CDI) on the inter-annual variation of NDVI (3). NDVI of eastern and south-eastern parts of the region were positively correlated with TMP (controlling PRE) (Fig 3A), whereas, the rest of the region displayed positive correlation with PRE (controlling TMP, Fig 3B). Conversely, negative correlation with TMP (controlling PRE) were identified for the western part of the region. This negative correlation reveals the effects of drought had combined with increased TMP and PET that resulted in restricted vegetation growth.

The analysis also reveals that elevation has an influence on which climate variables were important controls on NDVI. NDVI in low elevations in the eastern and southern parts of the region were negatively correlated with TMX (controlling TMN), but were positively correlated with TMN (controlling TMX) (Fig 3C and 3D). NDVI in the higher elevations in the central part of the region were positively correlated with TMX (controlling TMN), but negatively correlated with TMN (controlling TMX). Partial correlation between WMI and NDVI (Fig 3E, controlling CDI) displayed similar spatial pattern with Fig 3A, suggesting the temperature variations below the threshold of 5°C is less important in controlling the vegetation growth. The insignificant role of coldness on controlling the vegetation growth was confirmed by non-significant partial correlation coefficients between CDI and NDVI (controlling WMI, Fig 3F).

## Discussion

### Characteristics of climate change

This study expanded on the traditional understanding of regional climate warming, it also revealed asymmetric climate warming characterized by higher trend in TMN than TMX. This disparity results in decreased diurnal temperature range (TMX–TMN) [12,36]. As the daily temperature minima is influenced by night time net radiation, increased TMN was likely caused by increased night time cloud coverage. This observation coincides well with the positive trend of CLD found in this study (Table 2). The increase in CDI also coincided with winter warming, which has been widely reported [37]. The thermal change points that took place in 1996 coupled with decreased CDI during post-1996 period coincide with the recovery of Siberian High intensity [38,39]. The significant change point of temperature-related variables may also be related to 1997/1998 El Niño event [40–43].

This study revealed a positive but nonsignificant trend of PRE which is coincided with previous studies within the Loess Plateau [13] and in Northwest China [17]. Increased PRE may have also driven increased actual ET through vegetation transpiration [21,44]. However, the benefits of positive PRE trend are negated by significant rising PET, which was largely thermically driven. Higher PET caused the regional climate to shift towards warming and drying trends. Consequently, the regional climate change could be summarized as warming and drying and influenced by larger scale climate dynamics such as the El Niño, Southern Oscillation and the Siberian High intensity.

### Vegetation dynamics

Over the past three decades, numerous national and local ecological restoration projects had been implemented by the central and local governments of China. Examples of these national projects include the Live-stocks Loading Balance and Award Program (starting from 2005), and the Fencing Grassland and Moving Users project (enforced in 2002), Three North Shelter Forest System Project (starting from 1979), the Grain for Green Program (starting from 1999), and the Natural Forest Conservation Program (starting from 2000), in order to facilitate ecological security [19,45] and sustainable development [24,46], resulting in the positive trends of vegetation growth and regional actual evapotranspiration [21]. As a result of high variance of NDVI in Ordos [47], annual scale climate variables displayed non-significant correlation with NDVI (Table 3), which suggests there is a weak link between climate change and vegetation dynamics over the past three decades [23]. Similarly, R^2^ values of the principal regression analysis were low (Fig 2D), suggesting the limited contribution of inter-annual climate variability on vegetation dynamics. Previous studies also reported that the regional NDVI displayed opposite trend with the prediction using climate variables as model inputs [23,48]. Moreover, the vegetation resilience and the spatial distribution of vegetation types were accountable for the non-significant inter-annual climate-vegetation relationship (Table 5). For example, half of the plateau displayed positive correlation between precipitation and NDVI, whereas, weak correlation coefficients were mainly distributed in Hobq Desert and the Mu Us Sandy Land [22].

**Table 5:** Available studies on regional vegetation dynamics and its controlling factors.

Authors	Temporal Span	Data Sources	Vegetation Trend	Controlling Factors	Human effects
Ma et al. [22]	2000–2016	MODIS MOD13Q1	Greening	Humidity	Positive
Zhang et al. [19]	2001–2014	MODIS MCD12Q1	Positive	Co-work of climate and human activities	Positive
Zhou et al. [48]	1993–2000	GIMMS ndvi3g	Negative	Afforestation, Husbandry feeding reform	Positive
Fang et al. [49]	1982–2000	Landsat-5 TM GIMMS ndvi3g	Positive	Geologic structure	-
Dong et al. [50]	2000–2010	Landsat TM	Expanded forest land and farmland, reduced grassland	Humidity, Grain to Green Project and Road Density	Positive
Liu & Xin. [51]	2000–2017	MODIS MOD13A2	Positive	Humidity and Human activities	Positive
Sun et al. [52]	2000–2019	MODIS NDVI	Positive	Climatic factors and Afforestation	Positive

Besides, some edaphic and topographic factors were accountable for the spatial distribution of NDVI [53,54]. For example, the lithology of bedrock greatly affects vegetation cover and distribution in the Mu Us Sandy Land area, whereas, a high percentage farmlands and grasslands with large NDVI values are mainly distributed on low-permeability strata, such as the Quaternary Lake and alluvial deposits [49]. Moreover, it is also likely that vegetation dynamics may links to depletion of ground water [21], possibly leading to the rapid loss of regional lakes [55].

To further address the influence of human activity on climate-vegetation relationship, two typical pixels with negative NDVI slope were selected. Land use change from 1984 to 2015 at the 1st pixel (39°41′50.6″N, 109°56′20.45″E) displays the expansion of urban land and the 2nd pixels were related to lake extinctions (40°09′44.46″N, 108°27′28.04″E). In addition, the Global Human Influence Index Dataset of the Last of the Wild Project, Version 2, 2005 (LWP-2), was used as a surrogate for human activities (data not shown). This dataset derived from nine global data layers covering human population pressure (population density), human land use and infrastructure (built-up areas, nighttime lights, land use/land cover), and human access (coastlines, roads, railroads, navigable rivers) [56] and had been applied to study the influence of human activity on species distribution, biodiversity protection and vegetation activities [15,57]. Preliminary investigation on the correlation between the the spatial distribution of human influence index and NDVI trends suggests the positive role of human influences. Further studies are needed to address the detailed information of the influence of human activity on vegetation growth and dynamics.

## Supporting information

S1 Fig The raw data for trend analysis in Fig 1 and Table 2. (TIF)

S2 Fig The raster files for building Fig 2. (RAR)

S3 Fig The raster files for building Fig 3. (RAR)
