# Supplementary figures and images for: Inter-annual variations of vegetation dynamics to climate change in Ordos, Inner Mongolia, China
Source: PLoS One. 2022 Nov 4;17(11):e0264263. doi: 10.1371/journal.pone.0264263 (PMC9635713; doi:10.1371/journal.pone.0264263)

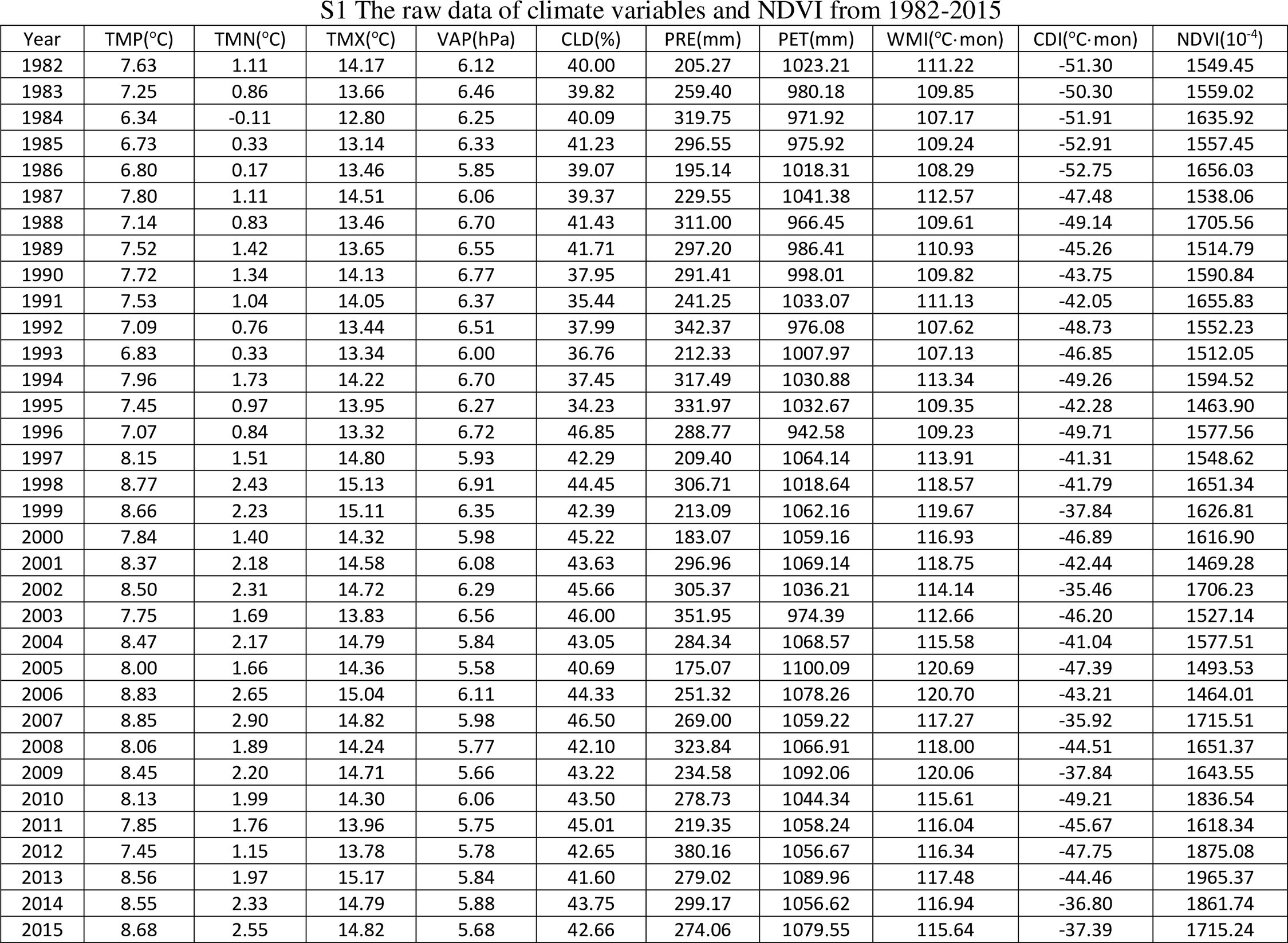

Supplement: S1 Fig — (TIF) [file pone.0264263.s001.tif]
